# 超高效液相色谱-串联质谱法同时测定化妆品中83种糖皮质激素

**DOI:** 10.3724/SP.J.1123.2023.04009

**Published:** 2023-12-08

**Authors:** Qianru ZHAO, Hua LIU, Yaping MENG, Xiang LI, Ruifang GAO, Xiangsheng LI

**Affiliations:** 安徽省食品药品检验研究院,安徽合肥230051; Anhui Institute for Food and Drug Control, Hefei 230051, China

**Keywords:** 超高效液相色谱-串联质谱, 糖皮质激素, 化妆品, 非法添加, ultra performance liquid chromatography-tandem mass spectrometry (UPLC-MS/MS), glucocorticoids, cosmetics, illegal addition

## Abstract

近年来化妆品中非法添加糖皮质激素的恶性事件常有发生,因此建立一个高通量且简便高效的检测方法,对保障消费者的用妆安全具有现实意义。研究建立了超高效液相色谱-串联质谱法同时测定化妆品中83种糖皮质激素的分析方法。以化妆品中常用的水、乳液、膏霜(o/w型)3种基质为研究对象,对样品前处理和色谱-质谱条件进行优化,最终确定样品经乙腈涡旋分散、超声提取、过滤后,采用Thermo Accucore PFP色谱柱(100 mm×2.1 mm, 2.6 μm)分离,以0.1%(v/v)乙酸乙腈和0.1%(v/v)乙酸水溶液为流动相梯度洗脱,在电喷雾正离子模式(ESI^+^)下以动态多反应监测(MRM)方式测定,外标法定量。结果表明,83种糖皮质激素在2~200 μg/L范围内线性关系良好,相关系数(*r*)均大于0.995,在3个不同添加水平下,平均回收率为74.5%~112.4%,相对标准偏差(RSD, *n*=6)为0.8%~9.9%,方法的检出限(LOD, *S/N*≥3)和定量限(LOQ, *S/N*≥10)分别为0.001~0.023 μg/g和0.002~0.076 μg/g。采用该方法对市售的41批化妆品进行83种糖皮质激素筛查,共发现4批阳性样品,分别检出氟轻松、倍氯米松双丙酸酯、醋酸地索奈德及地索奈德,含量范围为0.53~634.27 μg/g,其中醋酸地索奈德尚未列入法定检测标准的筛查范畴。该方法简便快捷,灵敏度高,重复性好,是对现行检测标准的补充,适用于多种基质类型化妆品中83种糖皮质激素的快速定性定量筛查,为化妆品质量的日常监督提供可靠的技术支持。

## Abstract (en)

Glucocorticoids, which are a class of steroidal hormones secreted by the adrenal cortex, have significant anti-inflammatory, immunosuppressive, and anti-allergic effects. Thus, these compounds are widely used in clinical practice. However, the long-term use of cosmetics containing glucocorticoids can lead to serious consequences, such as hormone-dependent dermatitis, hypertension, and other serious injuries. The Safety and Technical Specification for Cosmetics (2015 edition) and Regulation (EC) No. 1223/2009 of the European Parliament and Council on cosmetic products list glucocorticoids as prohibited raw materials. According to the National Medical Products Administration, reports on the illegal addition of glucocorticoids to cosmetics by manufacturers have increased in recent years. Therefore, establishing high-throughput screening methods to ensure the quality and safety of cosmetics is imperative. In this study, a comprehensive analytical method based on ultra performance liquid chromatography-tandem mass spectrometry (UPLC-MS/MS) was developed for the rapid screening of 83 glucocorticoids in cosmetics. A series of conditions were optimized using three matrices that are commonly used in cosmetics: water, lotion, and cream (o/w-type). Four mobile-phase systems and three chromatographic columns were then optimized to achieve the best separation effects. Various MS parameters, such as the capillary voltages, cone voltages, desolvation gas flow rates, and collision energies of the ion pairs of the target compounds, were also optimized. Furthermore, pretreatment was essential for glucocorticoid determination owing to the complex matrix effects of cosmetics. The analytes were divided into two groups, with lg *K*_ow_=4 as the limit, to compare the effects of the extraction solvent on recoveries. The extraction recoveries of target analytes with six extraction methods, namely, extraction with acetonitrile, extraction with acetone, extraction with ethyl acetate, dispersion in saturated sodium chloride solution followed by extraction with acetonitrile, dispersion in saturated sodium chloride solution followed by extraction with acetone, and dispersion in saturated sodium chloride solution followed by extraction with ethyl acetate, were compared. The recoveries from QuEChERS and solid-phase extraction (SPE) purification were also compared. Based on the experimental results, the final sample pretreatment method included acetonitrile vortex dispersion, ultrasonic extraction, and sample loading after filtration. The 83 target compounds were separated on a Thermo Accucore PFP column (100 mm×2.1 mm, 2.6 μm) with 0.1% (v/v) acetic acid in acetonitrile and 0.1% (v/v) acetic acid in water as the mobile phases. The analytes were determined by dynamic multiple-reaction monitoring (MRM) in electrospray positive ionization mode (ESI^+^) and quantified using the external standard method. Matrix standard curves were used to reduce matrix effects. The calibration curves of the 83 target compounds were linear in the mass concentration range of 2-200 μg/L (*r*>0.995). At three levels of addition, the recoveries were 74.5%-112.4%, and the relative standard deviations (RSDs, *n*=6) were 0.8%-9.9%. The limits of detection (LODs, *S/N*≥3) were 0.001-0.023 μg/g, and the limits of quantification (LOQs, *S/N*≥10) were 0.002-0.076 μg/g. The developed method was used to detect glucocorticoids in 41 cosmetic samples. Fluocinolone acetonide, beclomethasone dipropionate, desonide 21-acetate, and desonide were detected in four samples. The content range of glucocorticoids in the positive samples was 0.53-634.27 μg/g. Notably, desonide 21-acetate, which is not included in the scope of the statutory detection method, was detected in two batches of samples. In conclusion, the proposed method is simple, sensitive, reliable, and suitable for the high-throughput analysis of the 83 glucocorticoids in cosmetics with different matrices. This method could provide reliable technical support for the daily supervision of cosmetics and serve as a supplement to current glucocorticoid standards.

糖皮质激素为一类由肾上腺皮质中束状带分泌的甾体激素,具有抗炎作用,是皮肤科的常用药物^[1]^。长期使用糖皮质激素可能导致激素依赖性皮炎、柯兴综合征等不良反应,甚至引起高血压、糖尿病、骨质疏松症等严重损伤^[2⇓-4]^。我国《化妆品安全技术规范》(2015年版)^[5]^及欧盟《化妆品法规1223/2009》^[6]^等都将糖皮质激素列为禁用原料,某些商家为了牟利在化妆品中非法添加糖皮质激素,以期快速实现美白、祛斑、消炎等效果,因此导致“激素脸”、“大头娃娃”等恶性事件的发生^[7,8]^。更为严重的是,不法商家为了逃避监管往往非法添加法定检验方法以外的激素,为了更好地保障消费者权益,亟需建立种类更加全面的激素检测方法,为监管部门提供技术支撑。

近年来关于化妆品中糖皮质激素的检测方法主要有高效液相色谱法(HPLC)^[9]^、超高效液相色谱-高分辨质谱法(UPLC-HRMS)^[10⇓-12]^、超高效液相色谱-串联质谱法(UPLC-MS/MS)^[13⇓⇓⇓⇓⇓⇓⇓⇓⇓-23]^等。HPLC普及率高,但灵敏度不够且无法实现高通量的筛查,已经较少用于化妆品中禁用成分的检测。UPLC-HRMS分辨率高,但仪器昂贵,数据分析复杂,推广仍有难度^[24,25]^。UPLC-MS/MS灵敏度高,专属性强,且适用于高通量分析,是目前糖皮质激素检测的主流方法。目前报道的化妆品中糖皮质激素的前处理方法有固相萃取法^[13,14,16,20⇓-22]^、QuEChERS法^[15,19]^、溶剂分散提取法^[5,10⇓-12,17,18,23]^等,前两种方法可以在一定程度上减少化妆品复杂基质带来的干扰,但吸附剂在吸附杂质的同时也会吸附待测物,尤其当待测组分较多时,难以满足所有化合物的回收率要求,并且前处理复杂、耗时,检测效率低下。

现行已出台的化妆品中糖皮质激素检测的标准方法中,GB/T 24800.2-2009^[20]^包括41种糖皮质激素,SN/T 2533-2010^[21]^包括17种糖皮质激素,SN/T 4504-2016^[22]^包括3种糖皮质激素,已纳入《化妆品安全技术规范》(2015版)^[5]^的国家药品监督管理局2019年第66号通告包括50种糖皮质激素,GB/T 40145-2021^[23]^包括11种糖皮质激素。为了保障用妆安全,日常监督检验中需要对化妆品进行多种糖皮质激素的筛查,但上述各标准的前处理方法各异,样品需要处理多次再分别上机分析,耗时耗力,严重制约工作效率。本研究工作在前期调研的基础上,增加了部分现行标准检测范围外的糖皮质激素,并对前处理方法、质谱条件及色谱分离条件等进行优化,建立了UPLC-MS/MS同时检测化妆品中83种糖皮质激素的高通量检测方法,与现行检测方法相比,灵敏度更高,检测范围更广,且前处理更简单方便,有利于提高大批量筛查的工作效率,提高问题产品的发现率,更好地保障消费者的用妆安全,为化妆品日常监督提供可靠的技术支持。

## 1 实验部分

### 1.1 仪器、试剂与材料

ACQUITY^TM^ UPLC超高效液相色谱和Xevo TQ-S质谱仪(美国Waters公司); XP205电子分析天平(瑞士Mettler公司); Milli-Q全自动纯水机(美国Millipore公司);涡旋仪(德国IKA公司); TGL-20000cR高速台式冷冻离心机(上海安亭科学仪器厂); P180H超声仪(德国Elma公司)。

83种糖皮质激素标准品,纯度均大于96%,分别购自中国食品药品检定研究院、美国药典委员会、德国Dr. Ehrenstorfer公司和加拿大TRC公司。乙腈、乙酸(色谱纯,美国ThermoFisher公司);实验用水均为Milli-Q全自动纯水机制备的超纯水。

### 1.2 标准溶液的配制

分别精密称取83种糖皮质激素标准品适量(精确到0.01 mg),用乙腈溶解并定容至刻度,配制成1000 mg/L的单标准储备溶液,保存于-20 ℃冰箱。

分别精密吸取单标准储备液适量,用乙腈稀释至刻度,摇匀,配制成10 mg/L的混合标准储备溶液;精密吸取一定体积的混合标准储备溶液用基质空白提取溶液定容,配制2~200 μg/L的系列基质混合标准溶液。

### 1.3 样品处理

#### 1.3.1 样品前处理

精密称取0.2 g(精确到0.01 mg)均匀试样于10 mL聚丙烯离心管中,加入少量乙腈,于涡旋混合器上涡旋30 s使样品分散均匀,再加入乙腈至近刻度,超声提取20 min,静置至室温,用乙腈定容至刻度,摇匀,以10000 r/min离心5 min,经0.22 μm微孔滤膜过滤,滤液作为样品溶液。

#### 1.3.2 基质空白提取溶液

精密称取0.2 g(精确到0.01 mg)空白试样于10 mL聚丙烯离心管中,按照1.3.1节操作处理,即得基质空白提取溶液,用于配制系列基质混合标准溶液。

### 1.4 分析条件

色谱柱:Thermo Accucore PFP色谱柱(100 mm×2.1 mm, 2.6 μm);流动相A: 0.1%(v/v)乙酸乙腈,流动相B: 0.1%(v/v)乙酸水溶液;流速:0.3 mL/min;进样体积:1 μL;柱温:30 ℃;梯度洗脱程序:0~8 min, 26%A; 8~13 min, 26%A~28%A; 13~17 min, 28%A~30%A; 17~20 min, 30%A~38%A; 20~22 min, 38%A~42%A; 22~24 min, 42%A~55%A; 24~26 min, 55%A; 26~28 min, 55%A~80%A; 28~30 min, 80%A; 30~30.1, 80%A~26%A; 30.1~35 min, 26%A。

离子源:电喷雾离子源(ESI);扫描方式:正离子扫描;检测模式:多反应监测(MRM)模式;毛细管电压:2.5 kV;脱溶剂气流速:800 L/h;脱溶剂气温度:600 ℃;进样锥气体流速:100 L/h;离子源温度:150 ℃。其他质谱条件见表1。

**表1:** 83种糖皮质激素的保留时间、质谱参数和lg *K*_ow_值

No.		Analyte	*t*_R_/ min		Parent ion (*m/z*)		Product ions (*m/z*)		Cone voltage/V		Collision energies/eV				lg *K*_ow_	
1		triamcinolone (曲安西龙)	4.07		395.2		375.2^*^/357.2		2		6/		12		0.83	
2		prednisolone (泼尼松龙)	5.69		361.2		147.1^*^/325.2		4		28/		8		1.50	
3		hydrocortisone (氢化可的松)	5.68		363.2		121.1^*^/327.2		30		24/		16		1.43	
4		prednisone (泼尼松)	7.10		359.2		313.2^*^/147.1		34		10/		24		1.57	
5		cortisone (可的松)	6.79		361.2		163.1^*^/121.1		10		22/		32		1.44	
6		methylprednisolone (甲基泼尼松龙)	8.99		375.2		161.1^*^/339.2		18		18/		6		1.99	
7		betamethasone (倍他米松)	9.68		393.2		373.2^*^/355.2		10		4/		10		1.87	
8		dexamethasone (地塞米松)	10.29		393.2		355.2^*^/337.2		2		10/		12		1.87	
9		flumethasone (氟米松)	13.14		411.2		253.2^*^/121.1		28		14/		36		1.84	
10		beclomethasone (倍氯米松)	24.14		409.2		373.2^*^/147.0		12		10/		32		4.59	
11		triamcinolone acetonide (曲安奈德)	17.60		435.2		397.2^*^/339.2		4		14/		14		2.50	
12		fludroxycortide (氟氢缩松)	16.84		437.3		91.1^*^/105.1		4		62/		44		2.50	
13		triamcinolone diacetate (曲安西龙双醋酸酯)	19.11		479.3		441.2^*^/321.2		6		10/		14		2.16	
14		prednisolone 21-acetate (泼尼松龙醋酸酯)	15.29		403.2		147.1^*^/325.2		4		22/		10		2.58	
15		fluoromethalone (氟米龙)	15.65		377.2		279.2^*^/339.2		32		14/		10		2.02	
16		hydrocortisone 21-acetate (氢化可的松醋酸酯)	14.86		405.3		309.2^*^/121.1		2		14/		30		2.51	
17		deflazacort (地夫可特)	19.20		442.2		142.0^*^/124.0		68		36/		44		2.02	
18		fludrocortisone 21-acetate (氟氢可的松醋酸酯)	17.23		423.2		239.2^*^/325.2		64		24/		20		2.32	
19		prednisone 21-acetate (泼尼松醋酸酯)	20.03		401.2		295.2^*^/147.1		6		14/		24		2.66	
20		cortisone 21-acetate (可的松醋酸酯)	19.40		403.2		163.1^*^/343.2		2		26/		18		2.53	
21		methylprednisolone 21-acetate (甲基泼尼松龙醋酸酯)	20.63		417.3		339.2^*^/321.2		12		8/		12		3.08	
22		betamethasone 21-acetate (倍他米松醋酸酯)	21.51		435.2		397.2^*^/319.2		2		8/		12		2.96	
23		budesonide (布地奈德)	22.15		431.3		147.1^*^/323.2		2		28/		10		3.14	
24		hydrocortisone 17-butyrate (氢化可的松丁酸酯)	22.14		433.3		345.2^*^/121.1		26		12/		28		2.81	
25		dexamethasone 21-acetate (地塞米松醋酸酯)	21.91		435.2		397.2^*^/337.2		24		10/		10		2.96	
26		fluorometholone 17-acetate (氟米龙醋酸酯)	24.00		419.2		279.2^*^/321.2		2		14/		12		2.57	
27		hydrocortisone 17-valerate (氢化可的松戊酸酯)	24.09		447.3		345.2^*^/121.0		2		12/		28		3.52	
28		triamcinolone acetonide 21-acetate (曲安奈德醋酸酯)	24.70		477.3		339.2^*^/439.2		4		14/		14		3.61	
29		fluocinonide (氟轻松醋酸酯)	25.18		495.2		337.2^*^/121.1		2		16/		40		3.36	
30		diflorasone diacetate (二氟拉松双醋酸酯)	25.59		495.2		317.2^*^/279.2		24		12/		14		3.10	
31		betamethasone 17-valerate (倍他米松戊酸酯)	25.26		477.3		355.2^*^/279.2		4		16/		14		3.78	
32		prednicarbate (泼尼卡酯)	25.88		489.3		381.2^*^/115.0		22		8/		16		4.02	
33		halcinonide (哈西奈德)	25.52		455.2		359.2^*^/105.1		70		22/		58		3.31	
34		alclomethasone dipropionate (阿氯米松双丙酸酯)	26.26		521.3		301.1^*^/319.2		10		16/		14		3.94	
35	amcinonide (安西奈德)			25.88		503.3		399.2^*^/339.2		2		10/		14		3.52
36	clobetasol 17-propionate (氯倍他索丙酸酯)			26.48		467.2		373.1^*^/355.1		2		10/		14		3.98
37	fluticasone propionate (氟替卡松丙酸酯)			27.63		501.2		313.2^*^/293.2		38		10/		14		3.73
38	mometasone furoate (莫米他松糠酸酯)			26.85		521.2		355.1^*^/263.2		6		16/		22		4.27
39	betamethasone dipropionate (倍他米松双丙酸酯)			26.91		505.2		279.1^*^/319.2		6		16/		14		4.42
40	beclomethasone dipropionate (倍氯米松双丙酸酯)			27.31		521.3		319.2^*^/411.2		6		14/		10		4.59
41	clobetasone 17-butyrate (氯倍他松丁酸酯)			28.40		479.2		343.1^*^/279.1		8		16/		18		4.82
42	fluocinolone acetonide (氟轻松)			19.73		453.2		121.1^*^/433.3		26		34/		8		2.24
43	flumethasone 21-pivalate (双氟美松叔戊酸酯)			26.35		495.2		477.5^*^/253.1		18		8/		16		3.44
44	diflucortolone (双氟可龙)			20.75		395.1		355.2^*^/121.0		42		10/		30		2.72
45	fluticasone (氟替卡松)			24.97		445.1		405.1^*^/121.1		24		10/		40		3.73
46	clocortolone pivalate (新戊酸氯可托龙)			28.16		495.3		457.2^*^/337.1		32		10/		12		4.36
47	mometasone (莫美他松)			24.14		427.0		237.1^*^/373.1		30		22/		14		4.86
48	9-fluoro-16*α*,17-(isopropylidenedioxy) corticosterone			16.24		437.1		105.1^*^/121.1		62		52/		36		2.36
	(氢化曲安奈德)															
49	prednisolone valerate acetate (泼尼瓦酯)			26.71		487.2		367.1^*^/289.1		4		10/		14		4.58
50	fluprednisolone (氟泼尼龙)			6.69		379.1		341.1^*^/171.1		10		8/		28		1.51
51	deprodone (迪普罗酮)			10.54		345.1		309.1^*^/147.0		20		8/		22		3.55
52	isoflupredone acetate (9-氟泼尼松龙醋酸酯)			16.94		421.2		383.2^*^/295.2		26		8/		12		2.46
53	hydrocortisone hemisuccinate hydrate (氢化可的松半琥酯)			12.24		463.2		327.2^*^/309.2		8		12/		14		2.13
54	isoflupredone (异氟泼尼龙)			6.12		379.2		341.2^*^/237.1		24		10/		18		1.38
55	methylprednisolone aceponate (甲基泼尼松龙乙丙酸酯)			25.77		473.1		381.2^*^/321.1		18		8/		14		4.01
56	diflucortolone valerate (双氟可龙戊酸酯)			28.02		479.2		355.2^*^/375.2		6		14/		12		4.65
57	flumethasone acetate (双氟美松醋酸酯)			22.78		453.2		253.1^*^/433.2		48		18/		8		2.41
58	fludrocortisone (氟氢可的松)			6.43		381.2		239.1^*^/181.1		12		22/		30		1.23
59	halometasone (卤米松)			21.14		445.1		155.0^*^/287.1		8		32/		10		2.33
60	paramethasone (帕拉米松)			11.27		393.2		337.2^*^/355.2		30		10/		30		1.75
61	desonide 21-acetate (醋酸地索奈德)			24.63		459.2		323.2^*^/147.0		2		14/		34		2.89
62	ciclesonide (环索奈德)			28.82		541.3		323.2^*^/147.0		40		16/		42		4.70
63	desonide (地索奈德)			15.62		417.2		323.2^*^/147.0		12		10/		30		2.62
64	flunisolide (氟尼缩松)			20.71		435.2		321.1^*^/339.1		14		12/		14		2.27
65	desoximetasone (去羟米松)			17.41		377.3		339.2^*^/357.2		4		12/		10		2.20
66	halobetasol propionate (卤贝他索丙酸酯)			27.11		485.2		391.1^*^/121.0		28		10/		34		4.05
67	epi hydrocortisone 21-acetate (表氢化可的松醋酸酯)			11.53		405.1		241.0^*^/105.0		60		12/		28		2.35
68	fluocortolone (氟可龙)			18.99		377.2		303.2^*^/321.2		4		14/		8		1.86
69	methylprednisolone hemisuccinate (琥珀酸甲泼尼龙)			17.81		475.3		321.2^*^/339.2		20		12/		12		2.69
70	16*α*-hydroxyprednisolone (16a-羟基泼尼松龙)			3.75		377.2		323.2^*^/147.0		6		8/		24		0.95
71	loteprednol etabonate (氯替泼诺)			26.06		467.1		359.2^*^/265.1		22		10/		18		3.17
72	diflorasone (二氟拉松)			12.07		411.2		371.2^*^/253.1		2		10/		16		1.61
73	16*α*-hydroxy prednisolone acetate (16*α*-羟基泼尼松龙醋酸酯)			9.64		419.2		323.2^*^/147.0		26		12/		14		1.09
74	paramethasone acetate (醋酸帕拉米松)			22.77		435.2		319.2^*^/337.2		8		10/		10		2.32
75	16-meprednisone acetate (16-甲基泼尼松龙醋酸酯)			22.96		415.2		309.2^*^/355.2		24		12/		10		3.40
76	meprednisone (甲基泼尼松)			11.35		373.2		147.0^*^/171.0		24		24/		30		2.06
77	prednisolone 21-trimethylacetate (泼尼松龙戊酸酯)			24.91		445.3		307.2^*^/325.2		10		14/		21		3.15
78	difluprednate (双氟泼尼酯)			26.34		509.3		303.2^*^/321.2		8		14/		14		3.67
79	ulobetasol (卤倍他索)			23.67		429.2		90.7^*^/121.0		2		52/		44		3.09
80	hydrocortisone cypionate (氢化可的松环戊丙酸酯)			26.84		487.4		78.9^*^/107.0		10		50/		32		4.30
81	6*α*-fluoro-isoflupredone (6*α*-氟-异氟泼尼龙)			8.38		397.2		253.1^*^/121.0		12		16/		34		1.12
82	triamcinolone hexacetonide (己曲安奈德)			28.24		533.4		71.0^*^/99.0		2		26/		26		4.41
83	rimexolone (利美索龙)			25.91		371.3		295.2^*^/121.0		2		10/		42		4.01

*Quantitative ion. lg *K*_ow_: *N*-octanol-water partition coefficient.

## 2 结果与讨论

### 2.1 质谱条件的优化

在电喷雾离子源下,分别灌注100 μg/L的激素标准溶液,用正、负离子模式分别扫描,发现83种糖皮质激素均在正离子模式下响应较高。在正离子模式下,对待测物进行一级质谱全扫描,83种糖皮质激素的基峰离子均为准分子离子[M+H]^+^,再对待测物进行二级质谱扫描,选择两个特征碎片,并且优化碰撞能量,确定83种糖皮质激素的MRM参数,选择响应最大的碎片离子为定量离子,次级响应碎片离子为定性离子。优化后的质谱参数详见表1。

### 2.2 色谱条件的优化

糖皮质激素是以环戊多氢菲为母核的一系列化合物,83种待测化合物中有12组共32个化合物的相对分子质量相同,其中27个为同分异构体,母离子和子离子的质荷比(*m/z*)完全相同,单凭质谱不能实现定性判断,因此实现色谱分离是解决同分异构体准确定性并定量问题的关键。实验分别考察了Waters BEH C18(150 mm×2.1 mm, 1.7 μm)、Waters CSH C18(150 mm×2.1 mm, 1.7 μm)、Thermo Accucore PFP(100 mm×2.1 mm, 2.6 μm)3种色谱柱对分离的影响。前两种色谱柱对待测物均有较好的保留,但难以实现倍他米松、地塞米松等差向异构体的基线分离。Thermo Accucore PFP色谱柱(100 mm×2.1 mm, 2.6 μm)是以五氟苯基为固定相的色谱柱,苯环上的氟原子可以增强键合相和分析物之间的*π-π*相互作用、偶极-偶极作用及电荷转移作用,从而提高对待测物的保留和选择性,尤其对同分异构体有优异的选择性。结果显示,相较于前两种色谱柱,Thermo Accucore PFP色谱柱(100 mm×2.1 mm, 2.6 μm)可以明显提高同分异构体的分离度,因此本次实验选用Thermo Accucore PFP色谱柱(100 mm×2.1 mm, 2.6 μm)为分析柱。

在电喷雾正离子模式下,酸性环境可以提高待测物的质子化效率,从而提高响应,故在选定的色谱柱条件下,考察了0.1%(v/v)乙酸水溶液-乙腈、0.1%(v/v)乙酸水溶液-0.1%(v/v)乙酸乙腈、0.1%(v/v)甲酸水溶液-乙腈、0.1%(v/v)甲酸水溶液-0.1%(v/v)甲酸乙腈4种流动相体系,结果表明,0.1%(v/v)乙酸水溶液-0.1%(v/v)乙酸乙腈流动相体系下83种糖皮质激素均能获得较好的响应和峰形。通过对梯度洗脱程序的优化,可以实现所有同分异构体的基线分离,83种糖皮质激素的分布较为均匀。12组具有相同相对分子质量的糖皮质激素分离情况见图1。

**图1:**
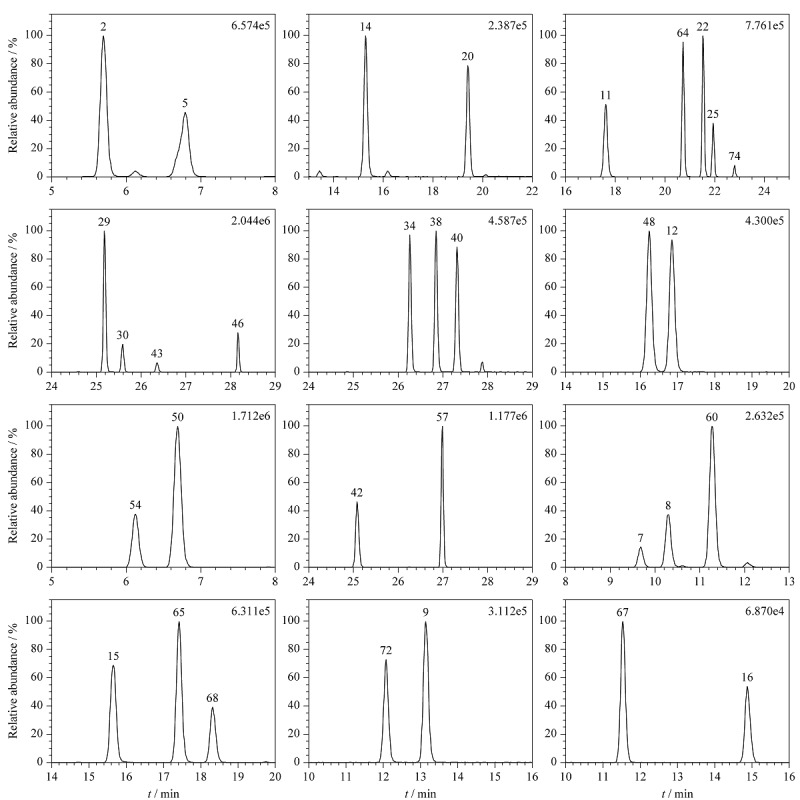
具有相同相对分子质量的12组糖皮质激素的色谱图

### 2.3 样品前处理条件的优化

目前化妆品中糖皮质激素的提取方法主要有超声提取法^[5,10⇓⇓-13,16⇓⇓-19,21⇓-23]^和溶剂萃取法^[12,14,15,20]^,提取溶剂普遍采用乙腈、甲醇等极性较强的溶剂,但本研究中涉及的糖皮质激素种类较多,83种糖皮质激素的lg *K*_ow_值为0.83~4.86(具体见表1),范围较宽,单一溶剂能否将所有组分提取完全有待考察。本实验以基质最复杂的膏霜(o/w型)为对象,将83种糖皮质激素以lg *K*_ow_=4为界限分成两组,分别比较了乙腈超声提取、丙酮超声提取、乙酸乙酯超声提取、饱和氯化钠分散后经乙腈提取、饱和氯化钠分散后经丙酮提取、饱和氯化钠分散后经乙酸乙酯提取6种不同提取溶剂对回收率的影响,结果显示对于lg *K*_ow_值小于4的糖皮质激素,乙腈为提取溶剂的峰形最好,随着提取溶剂的极性减弱,与流动相的相容性变差,曲安西龙、泼尼松龙等的溶剂效应严重;对于lg *K*_ow_值大于4的糖皮质激素,随着提取溶剂的极性减弱,少部分待测物的回收率略有提高,乙酸乙酯提取的回收率为70.4%~101.6%,丙酮提取的回收率为68.1%~101.2%,乙腈提取的回收率为77.1%~102.6%。综合考虑,最终选用乙腈超声提取83种糖皮质激素,样品只需前处理一次,节省溶剂且省时省力,更适合大批量样品的筛查。给出了lg *K*_ow_值大于4的16个待测物的提取回收率差异。

前期查阅文献和标准发现,目前化妆品中激素的检测方法大多采用对样品进行提取后再净化的前处理方式^[13⇓⇓-16,19⇓⇓-22]^,以减少化妆品复杂基质带来的干扰,但当待测组分较多时,净化难免会损失待测组分。为探索最佳的前处理方式,本实验以基质最复杂的膏霜(o/w型)为对象,在添加水平为0.2 μg/g的条件下,同时考察了固相萃取法(Oasis HLB固相萃取柱,3 mL/60 mg)、QuEChERS法(900 mg MgSO_4_+150 mg PSA+150 mg C_18_)和乙腈直接提取3种方式的提取回收率,结果表明,采用Oasis HLB固相萃取柱净化时,新戊酸氯可托龙、环索奈德、氢化可的松环戊丙酸酯的回收率较低,分别为42.3%、26.0%、47.0%,分析发现,洗脱溶液甲醇未能将这3种组分完全洗脱,究其原因,这3种糖皮质激素的极性较弱,甲醇的极性较强,无法将其完全洗脱,造成了回收率偏低;进一步比较了乙腈、甲醇、乙腈-甲醇(1∶1, v/v)3种洗脱液,结果无明显差异,表明固相萃取法不适用本法的样品净化;采用QuEChERS法处理样品时,氢化可的松半琥酯、琥珀酸甲泼尼龙的回收率较低,分别为22.9%、24.1%,经实验发现氢化可的松半琥酯、琥珀酸甲泼尼龙均含有羧基结构,为酸性化合物,PSA对其有吸附作用,而MgSO_4_在吸附水分的同时对这两种组分也有极强的吸附作用,造成回收率偏低,表明QuEChERS法也不适用本法的样品净化;采用乙腈直接提取的样品回收率为74.5%~112.4%,可以满足83种糖皮质激素的回收率要求。图3给出了10种典型糖皮质激素的提取回收率差异,综合考虑,本实验选择乙腈直接提取为样品的处理方式,既保证了提取回收率又简化了实验过程,且检测成本较低。

**图2:**
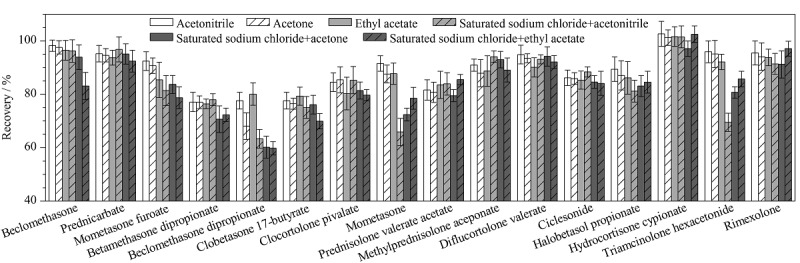
不同提取溶剂对16种lg *K*_ow_值大于4的糖皮质激素回收率的影响(*n*=6)

**图 3:**
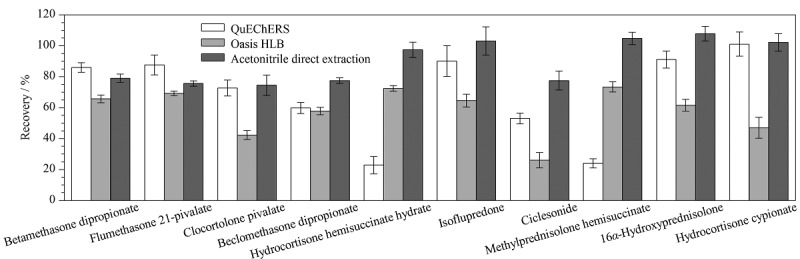
不同前处理方式对10种典型糖皮质激素回收率的影响(*n*=6)

### 2.4 基质效应(ME)评价

采用LC-MS/MS尤其是电喷雾模式分析时,待测物的离子化效率会受样品基质类型、前处理方法、仪器条件等因素的影响,因此使用LC-MS/MS须对基质效应进行评价,并采取适当的方式降低或者补偿基质效应带来的影响。本实验以基质最复杂的膏霜(o/w型)为考察对象,按照ME=(1-空白基质溶液中待测物的响应值/纯溶剂中待测物的响应值)×100%计算ME。当|ME|>50%时,表示存在较强的基质效应,当20%<|ME|≤50%时,表示基质效应中等,当|ME|≤20%时,表示基质效应较弱^[26]^。结果表明,40.96%(34/83)的糖皮质激素存在中等强度的基质效应。目前常用同位素内标、减少进样量、基质匹配标准曲线等方法来降低样品基质效应的影响,实现更准确的定性定量分析。其中基质匹配标准曲线法效果良好,且操作简单、经济实用^[27]^。因此本实验采用基质匹配标准曲线的方法降低基质效应的影响。

### 2.5 方法学评价

#### 2.5.1 线性范围、检出限和定量限

在优化的色谱、质谱条件下,对系列基质混合标准溶液进行测定,以定量离子峰面积为纵坐标,相应的质量浓度为横坐标绘制标准曲线,结果表明,83种糖皮质激素在2~200 μg/L范围内线性关系良好,相关系数(*r*)均大于0.995。逐级稀释系列基质混合标准溶液,以*S/N*≥3和*S/N*≥10确定83种糖皮质激素的检出限(LOD)和定量限(LOQ),分别为0.001~0.023 μg/g和0.002~0.076 μg/g。结果见表2。

**表2:** 83种糖皮质激素的线性方程、相关系数、检出限、定量限、回收率、精密度和基质效应

No.	Linear equation	*r*	LOD/(μg/g)	LOQ/(μg/g)	Recoveries/% (*n*=6)	RSDs/% (*n*=6)	ME/%
1	*y*=1.06×10^3^*x*+2.34×10^3^	0.9962	0.009	0.030	99.4, 102.2, 101.7	6.2, 3.5, 5.4	9.95
2	*y*=4.28×10^3^*x*-2.50×10^3^	0.9957	0.006	0.020	103.0, 99.9, 100.2	5.5, 2.9, 5.6	19.73
3	*y*=1.38×10^4^*x*-1.06×10^4^	0.9968	0.001	0.003	105.4, 105.4, 104.1	3.4, 3.2, 4.0	20.84
4	*y*=6.85×10^3^*x*-3.28×10^3^	0.9987	0.005	0.017	96.2, 101.7, 101.2	2.3, 3.0, 2.3	10.80
5	*y*=2.26×10^4^*x*-8.00×10^3^	0.9973	0.002	0.007	95.0, 105.7, 106.4	3.0, 2.8, 2.4	12.38
6	*y*=3.15×10^3^*x*-2.22×10^3^	0.9980	0.003	0.010	100.3, 110.2, 106.9	2.6, 4.9, 1.6	14.78
7	*y*=4.13×10^3^*x*-8.46×10^3^	0.9981	0.010	0.033	98.8, 105.8, 110.0	2.6, 5.5, 2.0	19.25
8	*y*=2.80×10^3^*x*-1.65×10^3^	0.9983	0.006	0.020	97.6, 105.9, 112.3	3.0, 8.2, 1.8	14.77
9	*y*=6.59×10^3^*x*-9.97×10^3^	0.9960	0.002	0.007	99.0, 95.4, 94.9	2.3, 8.0, 1.3	15.94
10	*y*=1.45×10^3^*x*+3.11×10^3^	0.9998	0.001	0.003	97.0, 103.3, 111.3	3.7, 7.6, 2.1	20.13
11	*y*=5.19×10^3^*x*-8.46×10^3^	0.9988	0.002	0.007	100.4, 99.8, 104.5	4.9, 5.6, 3.4	8.52
12	*y*=3.46×10^3^*x*+1.47×10^3^	0.9989	0.004	0.013	105.6, 109.7, 103.6	3.3, 7.7, 2.5	12.46
13	*y*=6.18×10^3^*x*-5.73×10^3^	0.9973	0.002	0.007	101.4, 98.7, 88.8	6.3, 7.6, 3.3	22.16
14	*y*=4.33×10^3^*x*+1.91×10^3^	0.9971	0.003	0.010	91.2, 101.0, 104.2	5.2, 6.0, 4.1	16.60
15	*y*=4.24×10^3^*x*-1.20×10^3^	0.9981	0.002	0.007	104.6, 100.3, 100.4	5.2, 5.0, 2.8	13.26
16	*y*=1.17×10^4^*x*-1.08×10^4^	0.9980	0.005	0.017	102.4, 101.9, 98.7	5.3, 6.9, 3.4	18.23
17	*y*=1.99×10^4^*x*-1.88×10^4^	0.9987	0.001	0.003	89.6, 99.8, 90.4	4.8, 5.6, 2.9	4.37
18	*y*=5.23×10^3^*x*-2.78×10^3^	0.9972	0.003	0.010	92.8, 94.0, 88.8	4.2, 4.0, 2.6	14.04
19	*y*=1.15×10^4^*x*-1.15×10^4^	0.9978	0.002	0.007	103.8, 106.6, 104.0	6.9, 9.9, 4.2	24.82
20	*y*=2.52×10^4^*x*-1.20×10^4^	0.9970	0.001	0.003	105.4, 105.3, 100.5	7.4, 9.2, 2.4	28.22
21	*y*=5.28×10^3^*x*-2.96×10^3^	0.9981	0.009	0.030	102.4, 96.8, 94.4	5.5, 5.2, 2.4	25.13
22	*y*=4.97×10^3^*x*-3.30×10^2^	0.9983	0.005	0.017	93.0, 91.5, 90.2	5.5, 3.9, 1.6	22.34
23	*y*=5.08×10^3^*x*-1.50×10^3^	0.9971	0.001	0.003	96.0, 99.7, 100.0	4.2, 5.3, 1.6	19.28
24	*y*=1.38×10^4^*x*-5.82×10^3^	0.9968	0.001	0.002	103.2, 102.7, 98.5	4.1, 4.9, 1.7	16.34
25	*y*=4.89×10^3^*x*-7.30×10^3^	0.9974	0.005	0.017	102.8, 94.4, 91.8	7.8, 6.8, 2.1	22.18
26	*y*=3.33×10^4^*x*-7.33×10^3^	0.9981	0.001	0.003	98.0, 100.5, 97.1	6.1, 4.8, 1.4	14.97
27	*y*=1.96×10^4^*x*-7.77×10^3^	0.9972	0.001	0.003	95.6, 101.2, 93.2	5.1, 4.9, 2.8	17.04
28	*y*=1.18×10^4^*x*-4.86×10^2^	0.9970	0.001	0.003	98.2, 97.0, 88.5	3.7, 2.6, 2.0	15.81
29	*y*=9.34×10^3^*x*-4.55×10^3^	0.9976	0.001	0.003	98.2, 99.0, 88.5	3.1, 2.3, 1.8	20.43
30	*y*=1.15×10^4^*x*-2.40×10^3^	0.9963	0.001	0.003	97.8, 97.8, 85.5	5.4, 3.1, 3.4	17.82
31	*y*=1.98×10^3^*x*-5.08×10^2^	0.9974	0.001	0.003	97.6, 92.8, 89.7	5.5, 4.2, 2.1	18.61
32	*y*=1.54×10^4^*x*+2.07×10^3^	0.9964	0.001	0.003	100.0, 93.5, 78.1	5.0, 4.7, 6.0	20.40
33	*y*=4.52×10^3^*x*+7.06×10^2^	0.9962	0.001	0.003	95.0, 92.5, 88.5	5.1, 5.9, 5.1	11.06
34	*y*=6.36×10^3^*x*-1.95×10^3^	0.9954	0.002	0.007	78.5, 98.0, 79.8	5.4, 2.1, 4.4	18.56
35	*y*=1.29×10^4^*x*+1.96×10^3^	0.9967	0.001	0.003	95.8, 89.9, 82.6	3.5, 3.0, 1.8	21.08
36	*y*=1.38×10^4^*x*+3.87×10^2^	0.9968	0.002	0.007	85.8, 82.5, 80.2	6.3, 5.2, 1.8	20.19
37	*y*=1.83×10^4^*x*-1.25×10^4^	0.9951	0.002	0.007	85.6, 75.0, 86.0	5.1, 2.3, 6.3	28.10
38	*y*=5.57×10^3^*x*-6.29×10^3^	0.9956	0.007	0.023	77.8, 76.8, 81.5	5.4, 4.9, 3.1	22.88
39	*y*=9.24×10^3^*x*-4.82×10^3^	0.9952	0.001	0.003	75.4, 79.0, 74.5	3.5, 2.7, 1.7	14.77
40	*y*=6.05×10^3^*x*-7.46×10^3^	0.9958	0.001	0.003	79.8, 77.5, 78.9	6.9, 1.8, 7.9	29.92
41	*y*=1.30×10^4^*x*+2.91×10^2^	0.9958	0.001	0.003	98.4, 79.2, 78.4	7.0, 8.1, 9.9	27.47
42	*y*=9.06×10^2^*x*-1.40×10^3^	0.9951	0.001	0.003	99.0, 96.3, 93.3	4.9, 6.0, 2.3	29.28
43	*y*=4.36×10^3^*x*-2.50×10^3^	0.9974	0.012	0.040	76.4, 75.6, 81.4	2.1, 1.8, 1.4	20.30
44	*y*=2.49×10^4^*x*-1.04×10^4^	0.9972	0.007	0.023	100.4, 93.8, 100.1	6.1, 8.0, 1.3	28.48
45	*y*=1.22×10^4^*x*+6.19×10^3^	0.9989	0.009	0.030	95.8, 97.2, 96.0	5.1, 6.1, 2.0	15.98
46	*y*=4.74×10^3^*x*-1.06×10^3^	0.9967	0.005	0.016	78.7, 74.5, 75.8	5.8, 6.6, 8.3	23.25
47	*y*=3.44×10^3^*x*-2.02×10^3^	0.9995	0.019	0.063	90.0, 91.7, 91.2	4.1, 4.6, 1.9	17.81
48	*y*=3.44×10^3^*x*-5.47×10^3^	0.9950	0.018	0.059	104.2, 101.4, 103.4	4.8, 8.0, 2.5	11.41
49	*y*=1.32×10^4^*x*-4.77×10^3^	0.9964	0.005	0.017	76.8, 79.7, 78.4	5.6, 7.9, 4.0	25.62
50	*y*=1.42×10^4^*x*-5.82×10^3^	0.9976	0.004	0.013	97.0, 102.3, 92.9	3.8, 3.0, 2.3	12.89
51	*y*=2.37×10^3^*x*-2.49×10^3^	0.9988	0.016	0.053	97.0, 100.3, 92.9	2.2, 6.8, 1.6	-2.05
52	*y*=3.71×10^3^*x*-3.34×10^2^	0.9955	0.015	0.050	95.6, 97.7, 92.9	4.6, 3.9, 2.9	19.98
53	*y*=9.00×10^3^*x*-9.18×10^3^	0.9995	0.005	0.016	96.8, 97.4, 96.7	5.0, 5.0, 2.7	7.77
54	*y*=4.06×10^3^*x*-9.52×10^2^	0.9995	0.014	0.046	100.8, 103.1, 99.1	5.6, 9.2, 8.8	24.48
55	*y*=9.37×10^3^*x*-6.45×10^3^	0.9986	0.008	0.026	99.8, 92.6, 78.9	9.6, 3.5, 5.7	20.91
56	*y*=1.10×10^4^*x*-3.86×10^3^	0.9965	0.001	0.003	86.6, 79.2, 85.8	8.6, 3.3, 6.1	29.62
57	*y*=1.79×10^4^*x*+4.30×10^2^	0.9963	0.004	0.014	97.2, 95.5, 89.5	5.1, 4.1, 2.2	10.64
58	*y*=4.54×10^3^*x*+2.26×10^3^	0.9951	0.016	0.053	106.0, 100.9, 103.0	1.1, 7.5, 6.6	20.17
59	*y*=2.77×10^3^*x*-2.80×10^3^	0.9977	0.023	0.076	103.4, 96.6, 99.8	1.1, 6.1, 2.3	17.15
60	*y*=2.72×10^3^*x*+3.30×10^3^	0.9992	0.009	0.030	95.6, 98.0, 90.8	5.4, 8.5, 1.5	16.54
61	*y*=1.41×10^4^*x*-2.89×10^3^	0.9985	0.005	0.017	97.0, 95.8, 87.9	3.1, 2.8, 1.7	17.45
62	*y*=8.20×10^3^*x*-1.67×10^3^	0.9972	0.001	0.003	78.2, 77.5, 75.6	4.9, 6.1, 8.5	28.90
63	*y*=7.06×10^3^*x*-1.17×10^4^	0.9975	0.001	0.003	99.2, 99.7, 102.4	5.1, 6.2, 3.8	16.91
64	*y*=3.59×10^3^*x*-7.65×10^3^	0.9997	0.013	0.044	103.4, 98.6, 99.5	4.7, 7.2, 2.2	14.30
65	*y*=6.12×10^3^*x*-4.01×10^3^	0.9989	0.009	0.030	105.8, 108.3, 106.3	5.3, 6.1, 2.1	20.96
66	*y*=6.30×10^3^*x*+1.66×10^3^	0.9955	0.001	0.003	84.0, 74.8, 77.8	7.1, 2.2, 3.3	20.56
67	*y*=8.23×10^2^*x*+1.70×10^3^	0.9997	0.009	0.030	104.0, 101.7, 93.7	5.5, 7.0, 3.9	9.49
68	*y*=6.33×10^3^*x*-6.95×10^3^	0.9969	0.009	0.030	93.4, 97.4, 103.6	4.2, 7.2, 2.1	23.65
69	*y*=2.63×10^3^*x*-2.14×10^3^	0.9988	0.008	0.026	105.0, 104.8, 104.2	6.5, 4.0, 3.6	12.89
70	*y*=2.69×10^4^*x*+2.26×10^4^	0.9979	0.005	0.015	98.6, 107.8, 112.2	7.2, 4.7, 5.0	5.85
71	*y*=1.46×10^4^*x*-1.30×10^4^	0.9970	0.001	0.003	88.8, 84.3, 79.5	6.6, 3.8, 5.7	18.52
72	*y*=2.45×10^3^*x*-1.40×10^3^	0.9998	0.009	0.030	102.0, 100.3, 103.1	3.5, 8.4, 1.1	24.14
73	*y*=3.01×10^3^*x*+4.00×10^2^	0.9996	0.008	0.026	98.6, 107.7, 85.2	6.3, 6.6, 7.7	14.18
74	*y*=4.60×10^3^*x*-7.07×10^3^	0.9991	0.008	0.026	105.0, 97.1, 88.9	4.2, 5.5, 2.8	19.17
75	*y*=2.32×10^4^*x*-1.30×10^4^	0.9973	0.007	0.023	95.6, 104.4, 102.4	6.4, 6.6, 1.9	10.31
76	*y*=6.37×10^3^*x*-9.39×10^3^	0.9978	0.002	0.007	78.5, 84.3, 104.4	8.2, 1.8, 9.8	19.60
77	*y*=7.80×10^3^*x*-1.36×10^3^	0.9991	0.003	0.010	93.7, 103.1, 105.0	5.2, 2.0, 2.8	17.44
78	*y*=1.45×10^4^*x*-4.07×10^3^	0.9981	0.002	0.007	86.7, 91.2, 100.7	4.5, 5.3, 4.3	24.82
79	*y*=1.28×10^3^*x*-2.04×10^3^	0.9979	0.006	0.020	93.2, 106.7, 109.1	7.9, 7.4, 8.4	16.65
80	*y*=1.24×10^4^*x*-6.57×10^3^	0.9972	0.005	0.017	89.2, 102.2, 99.7	5.6, 5.7, 2.1	13.59
81	*y*=5.82×10^3^*x*-5.94×10^2^	0.9992	0.009	0.030	91.0, 107.2, 112.4	6.8, 5.0, 8.3	13.76
82	*y*=5.57×10^3^*x*+3.29×10^3^	0.9992	0.009	0.030	78.2, 89.7, 90.8	7.7, 1.0, 2.7	29.27
83	*y*=2.59×10^3^*x*-1.47×10^3^	0.9987	0.009	0.030	78.2, 79.6, 89.5	5.9, 7.4, 0.8	28.24

*y*: peak area; *x*: mass concentration, μg/L.

#### 2.5.2 回收率和精密度

取0.2 g空白样品,在低、中、高(分别为0.1、0.2、1 μg/g)3个添加水平下进行加标回收试验,每个水平平行测定6次,计算得到平均回收率为74.5%~112.4%,相对标准偏差为0.8%~9.9%(*n*=6)。具体结果见表2。

#### 2.5.3 实际样品测定

采用本法对41批市售化妆品进行83种糖皮质激素的筛查,结果共检出4批阳性样品,检出率达9.76%。其中1批检出氟轻松,含量为6.54 μg/g; 1批检出倍氯米松双丙酸酯,含量为1.74 μg/g; 2批检出醋酸地索奈德和地索奈德,醋酸地索奈德含量为0.53 μg/g和0.61 μg/g,地索奈德含量为560.85 μg/g和634.27 μg/g。本次检出的氟轻松、倍氯米松双丙酸酯及地索奈德在《化妆品安全技术规范》(2015版)中的检出限均为0.03 μg/g,本次检出含量均高于检出限,可判定为非法添加禁用组分,但醋酸地索奈德尚未列入法定检测标准,表明非法添加法定检测方法外的糖皮质激素的现象仍然存在,亟需制定筛查范围更广的标准,保障消费者的用妆安全。

## 3 结论

本研究建立了超高效液相色谱-串联质谱法同时测定化妆品中83种糖皮质激素的高通量方法。实验优化了色谱、质谱参数,实现了多组同分异构体的基线分离。采用乙腈直接提取的前处理方式,简单高效,大大提高了监督检验的工作效率,该方法可用于水、乳、膏霜(o/w型)基质化妆品中83种糖皮质激素的筛查确证和定量分析,是对现行《化妆品安全技术规范》(2015年版)中激素检测方法的补充,可为化妆品的日常监管提供快速、高效、灵敏可靠的高通量检测方法。
